# The granulocyte colony-stimulating factor produced during *Streptococcus suis* infection controls neutrophil recruitment in the blood without affecting bacterial clearance

**DOI:** 10.3389/fimmu.2024.1403789

**Published:** 2024-08-02

**Authors:** Marêva Bleuzé, Mélanie Lehoux, Jean-Philippe Auger, Marcelo Gottschalk, Mariela Segura

**Affiliations:** Research Group on Infectious Diseases in Production Animals (GREMIP) & Swine and Poultry Infectious Diseases Research Center (CRIPA), Faculty of Veterinary Medicine, University of Montreal, St-Hyacinthe, QC, Canada

**Keywords:** *Streptococcus suis*, neutrophils, granulocyte colony-stimulating factor (G-CSF), cytokines, systemic inflammation, infection, immune cell recruitment, bacteremia

## Abstract

*Streptococcus suis* causes diseases in pigs and has emerged as a zoonotic agent. When infected, the host develops an exacerbated inflammation that can lead to septic shock and meningitis. Although neutrophils greatly infiltrate the lesions, their dynamics during *S. suis* infection remain poorly described. Moreover, very few studies reported on the production and role of a key factor in the regulation of neutrophils: the colony-stimulating granulocyte factor (G-CSF). In this study, we characterized the G-CSF-neutrophil axis in the pathogenesis of *S. suis* induced disease. Using a mouse model of *S. suis* infection, we first evaluated the recruitment of neutrophils and their activation profile by flow cytometry. We found that infection provokes a massive neutrophil recruitment from the bone marrow to the blood and spleen. In both compartments, neutrophils displayed multiple activation markers. In parallel, we observed high systemic levels of G-CSF, with a peak of production coinciding with that of neutrophil recruitment. We then neutralized the effects of G-CSF and highlighted its role in the release of neutrophils from the bone marrow to the blood. However, it did not affect bacteremia nor the cytokine storm induced by *S. suis*. In conclusion, systemic G-CSF induces the release of neutrophils from the bone marrow to the blood, but its role in inflammation or bacterial clearance seems to be compensated by unknown factors. A better understanding of the role of neutrophils and inflammatory mediators could lead to better strategies for controlling the infection caused by *S. suis.*

## 1 Introduction

The pig pathogen *Streptococcus suis* causes economic losses to the swine industry and represents a threat for humans due to its zoonotic potential (1). Several serotypes were described over the years, but the serotype 2 is considered the most prevalent and virulent worldwide (2). When *S. suis* invades the host, it causes septic disease manifested by endocarditis, meningitis, septic shock, and sudden death (1). These clinical manifestations arise from an exacerbated inflammation caused by the bacteria: while *S. suis* stimulates innate immune defenses, their incapacity to clear the bacteria leads to an outburst detrimental for the host (1). In this regard, studying the early events of the innate immune response against *S. suis* remains essential to understanding the pathogenesis of the disease caused by this bacterium.

Among the key cell types of the early immune response against pathogens, neutrophils quickly reach the site of infection under the influence of inflammatory signals (3, 4). When neutrophils reach the injured tissue, they strive to destroy pathogens with their microbicidal functions that include phagocytosis, neutrophil extracellular trap (NET) release, oxygen burst, and degranulation of antimicrobial cargo (3). The important role of neutrophils for pathogen clearance was demonstrated in a variety of infection models, including during *S. suis* infection (5–10). To perform their effector functions, neutrophils undergo phenotypical changes that turn them into an “activated” state (11, 12). This state can be visualized by changes in surface marker expression, including an enhanced expression of the integrin CD11b, the shedding of the L-selectin CD62L and the modulation of the CXC motif chemokine receptor 2 (CXCR2) (11–13). Although some studies report neutrophil recruitment in the blood, the brain and the lesions during *S. suis* infection in various animal models (10, 14–19), gaps exist concerning the precise dynamic of recruitment, the origin and the phenotypic changes of neutrophils during the early stages of *S. suis*-induced inflammation.

As mentioned, inflammatory signals dictate neutrophil intervention during infections. Of these, the granulocyte colony-stimulating factor (G-CSF), a glycoprotein involved in neutrophil differentiation, mobilization and activation, is amongst the most crucial (20, 21). In steady-state, G-CSF levels remain low, while infection leads to its production in various models (22–25). Several studies demonstrated that G-CSF participates in clearance of a variety of pathogens (26–28). Although such study was not conducted in a *S. suis* model of infection, a report evocated that administration of G-CSF extends the survival time of piglets challenged with *S. suis* (29). A second study showed that murine dendritic cells and macrophages produce G-CSF in response to *S. suis* via surface protein recognition (30). These results suggest that G-CSF could be of importance to fight *S. suis* in the early stages of the infection. However, information remains limited on the production and role of G-CSF during *S. suis* infection *in vivo*. In this study, we hypothesized that neutrophils are quickly mobilized and activated during *S. suis* infection, and that the G-CSF controls neutrophil mobilization, thereby participating in early immune response, bacterial clearance and the resolution of the infection.

## 2 Materials and methods

### 2.1 Ethics statement

The study was carried out accordingly with the recommendations of the guidelines and policies of the Canadian Council on Animal Care and the principles set forth in the Guide for the Care and Use of Laboratory Animals. The protocols and procedures were approved by the Animal Welfare Committee of the University of Montreal (protocols numbers Rech-1399 and Rech-1523), including euthanasia to minimize animal suffering, which was applied throughout this study when animals were seriously affected since mortality was not an endpoint measurement. Mice had free access to water and food pellets as well as enrichment such as diverse materials to use as bedding alongside access to a refuge and a rubber toy for gnawing.

### 2.2 *S. suis* strains and growth conditions

The well-characterized and encapsulated strain P1/7 (serotype 2, sequence type 1) was used in this study. This strain was isolated from a pig with meningitis in the United Kingdom and its virulence was previously reported in experimental models of infection (31, 32). *S. suis* was grown overnight on Columbia agar plate supplemented with 5% sheep blood (Oxoid, Nepean, ON, Canada) at 37°C with 5% of CO_2_. Isolated colonies were used to inoculate 5 ml of Todd Hewitt broth (THB; Becton, Dickinson, Mississauga, ON, Canada) for 8 h. To prepare working solutions, 10 µl of a 10^-3^ dilution of the suspension were inoculated in 30 ml of THB for 16 h at 37°C with 5% of CO_2_. Bacteria were washed twice with pH 7.4 phosphate-buffered saline (PBS) and resuspended in THB at the appropriate dilution. To determine the bacterial concentration in CFU/ml, suspensions were diluted and plated on THB agar plates and colonies counted.

### 2.3 *S. suis* experimental mouse infections

Wild-type C57BL/6 mice (5 to 8 weeks of age) were obtained from Charles River Laboratories (Wilmington, MA, USA). Mice were housed under specific pathogen-free conditions in ventilated cages and had unlimited access to water and rodent chow. Mice were intraperitoneally infected with 1 ml of *S. suis* suspension containing 1 x 10^7^ CFU. In this model, *S. suis* quickly reaches the bloodstream and spreads causing a systemic inflammatory response, as observed in pig natural infection (31). Control mice (mock-infected) were injected with the same volume of vehicle solution (THB). Clinical signs were observed every 6 h daily to monitor for severe clinical signs of infection. After different times post-infection, mice were euthanized to collect blood by intracardiac puncture, spleen, liver and/or bone marrow. Blood samples were collected from living mice at different time points (see below) via the caudal vein of the tail (to evaluate bacteremia) or the submandibular vein (to quantify pro-inflammatory mediators).

### 2.4 Granulocyte colony-stimulating factor receptor blockade

To neutralize the effect of the G-CSF, we applied an anti-mouse G-CSF receptor (G-CSFR) antibody (Ch5E2-VR81-mIgG1κ; CSL Innovation, Parkville, Australia) as previously described (33). Briefly, mice were intraperitoneally injected with 0.2 ml of the anti-mouse G-CSFR antibody (100 µg per dose) or the corresponding isotype control (mouse IgG1κ; either from Bioxcell, Lebanon, NH, USA or from Biolegend, San Diego, CA, USA). The dose of anti-mouse G-CSFR antibody was selected in pre-trials (data not shown). Mice that were infected for 72 h received four doses of antibodies at day -1, day 0 prior to infection and day +1 and day +2 after infection. Mice that were infected for less than 24 h received two doses of antibodies at day -1 and day 0 prior to infection. Mice were infected with *S. suis* as described above.

### 2.5 Evaluation of bacteremia

Five microliters of blood were collected at 12 h, 24 h, 48 h and 72 h post-infection through the caudal tail vein of mice. Samples were appropriately diluted in PBS, plated on THB agar and colonies counted to assess the blood bacterial load in infected mice.

### 2.6 Measurement of pro-inflammatory mediators in serum and organs

The blood of infected mice was collected at different time points post-infection either by intracardiac puncture on euthanized mice or by the submandibular vein collection on living mice. Blood was collected in BD Microtainer™ serum separator tubes (BD Biosciences, Mississauga, ON, Canada) and centrifugated at 10,000 *g* for 2 min. The serum was collected at the surface of the gel and stored at -80°C until analysis. The spleen and the liver were homogenized using a POLYTRON PT 1200E system bundle (Kinematica, Lucerne, Switzerland) in an extraction buffer containing complete Mini, EDTA-free, protease inhibitor cocktail tablets (Roche Diagnostics GmbH, Mannheim, Germany), following the manufacturer’s instructions. The tibias of mice were flushed in 500 µl of extraction buffer. Homogenate supernatants were collected by centrifugation and stored at -80°C until analysis.

The concentrations of G-CSF, interleukin (IL)-6 and the C-X-C motif chemokine ligand 1 (CXCL1)/keratinocyte chemoattractant (KC), were determined by sandwich enzyme-linked immunosorbent assay (ELISA) using pair-matched antibodies from R&D Systems (Minneapolis, MN, USA). Cytokine measurements were performed according to the manufacturer’s recommendations, as previously described (34, 35). The concentrations of other cytokines and chemokines were measured using a Bio-Plex Pro assay (Bio-Rad, Mississauga, ON, Canada) according to the manufacturer’s instructions. The assay covered the G-CSF, the interferon-γ (IFN-γ), the IL-12p70, IL-6, C-C motif chemokine ligand 2 (CCL2)/MCP-1, CCL3/MIP-1α, CCL4/MIP-1β, CCL6/RANTES, CXCL9/MIG, and CXCL2/MIP-2. Acquisition was performed on the MAGPIX platform (Luminex, Toronto, ON, Canada), and data were analyzed using Bio-Plex Manager 6.1 software (Bio-Rad).

### 2.7 Flow cytometry analyses

Blood was collected in tubes coated with lithium heparin (BD Biosciences). Organs freshly collected from euthanized mice were homogenized to obtain single-cell suspensions. In the samples, red cells were removed by suspending the cells in a commercial lysis buffer (Invitrogen, Burlington, ON, Canada). Suspensions were first stained with a Fixable viability Dye eFluor™ 506 (Invitrogen) to exclude the dead cells from the analysis. After washing with PBS containing 2% fetal bovine serum, surface Fc receptors - that non-specifically bind antibodies - were blocked using FcBlock (BD Biosciences) for 15 min before staining. The cells were then stained for the markers of interest (detailed below) for 30 min in PBS containing 2% fetal bovine serum before being fixed by suspending them for 15-30 min in cold paraformaldehyde-PBS (2% for extracellular staining and 4% for intracellular staining) and stored in PBS at 4°C until data acquisition. To count the cells, 10 µL of Countbright™ Absolute Counting Beads (Invitrogen) were added in 400 µL of cell suspension just before acquisition. Data were acquired on a BD Fortessa or a BD FACSAria Fusion using the BD FACSDiva software and analyzed using the Flow Jo software (BD Biosciences).

The different subsets of cells were identified as previously described (36) using the following antibodies: PE-conjugated anti-mouse CD45 (clone 30-F11), APC-conjugated anti-mouse CD11c (clone HL3), BV421-conjugated anti-mouse CD11b (clone M1/70), FITC-conjugated anti-mouse Ly6G (clone 1A8). In addition to neutrophil markers, cells were stained with the following antibodies: PE CF594-conjugated anti-mouse CXCR4 (clone 2B11/CXCR4), BV605-conjugated anti-mouse CD49d (clone R1-2), Alexa700-conjugated anti-mouse CD62L (clone MEL-14), BV711-conjugated anti-mouse CXCR2 (CD182) (clone V48-2310). For BV-conjugated antibodies, the BD Horizon™ Brilliant Stain Buffer was used. To stain intracellular G-CSF, cells were permeabilized by suspension in BD Perm/Wash™ Buffer for 15 min in the dark and stained with AlexaFluor700-conjugated anti-mouse G-CSF antibody (clone 67604; R&D Systems). Unless otherwise indicated, all antibodies and buffers were from BD Biosciences. The gating strategy is depicted in **Supplementary Figure S1**.

### 2.8 Statistical analysis

Experiments were repeated at least three times, and the number of animals varied from three to thirty-six depending on the condition or time point (see figure legends for details). For all data, the normality of distribution was verified using the Shapiro-Wilk test. Accordingly, parametric tests (unpaired *t* test) or nonparametric tests (Mann-Whitney rank sum test) were performed to evaluate statistical differences between groups. Data are presented as the mean +/- standard error of the mean (SEM). *P* < 0.05 was considered statistically significant.

## 3 Results

### 3.1 *S. suis* infection induces changes in immune cell populations

**Figure 1:**
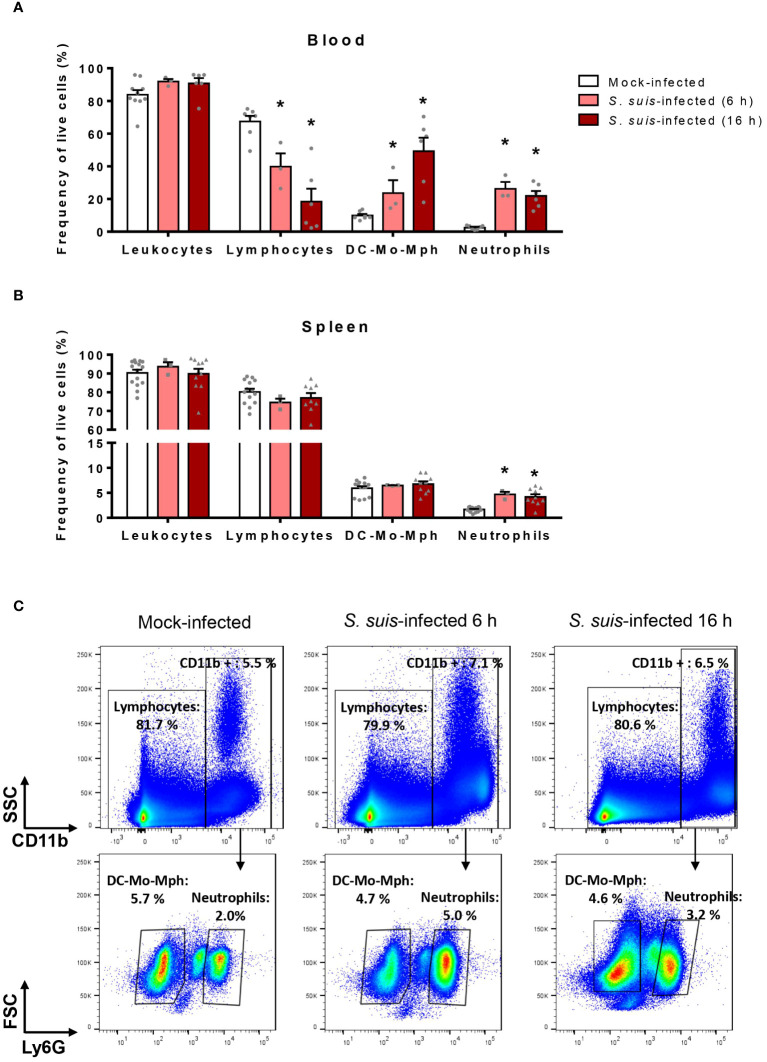
*S. suis* infection induces changes in immune cell populations. Mice were intraperitoneally infected with *S. suis* serotype 2 (1 x 10^7^ CFU/ml) or mock-infected. After 6 h or 16 h of infection, blood **(A)** and spleen **(B)** were collected and cells analyzed by flow cytometry. DC-Mo-Mph stands for the pool of dendritic cells, monocytes and macrophages. Data are expressed as the percentage of each cell population among live cells in the blood **(A)** or in the spleen **(B)** expressed as mean +/- SEM (*n =* 3 to 10). *P < 0.05, indicates a significant difference between mock-infected and *S. suis-*infected mice. **(C)** Representative dot plots illustrating the gating strategy used to identify spleen immune cells in mock-infected (left) or *S. suis*-infected animals for 6 h (middle) and 16 h (right).

*S. suis* triggers an exaggerated inflammatory response in the host, including the production of multiple chemokines (31). Since those molecules participate in immune cell migration (37), we evaluated the changes in immune cell populations in the blood and the spleen at the early stages of *S. suis* infection. The markers and gating strategy allowed to distinguish leukocytes, lymphocytes, neutrophils and the pool of dendritic cells (DC), monocytes (Mo) and macrophages (Mph) (**Supplementary Figure S1**). During *S. suis* infection, the percentage of neutrophils significantly increased by 10-fold in the blood and by 3-fold in the spleen (**Figures 1A, B**). Other changes occurred in the blood: the percentage of lymphocytes decreased while the percentage of the pool DC-Mo-Mph increased (**Figure 1A**). However, no changes of these two cell populations were observed in the spleen (**Figures 1B, C**).

### 3.2 Neutrophils migrate from the bone marrow to the blood during *S. suis* infection

**Figure 2:**
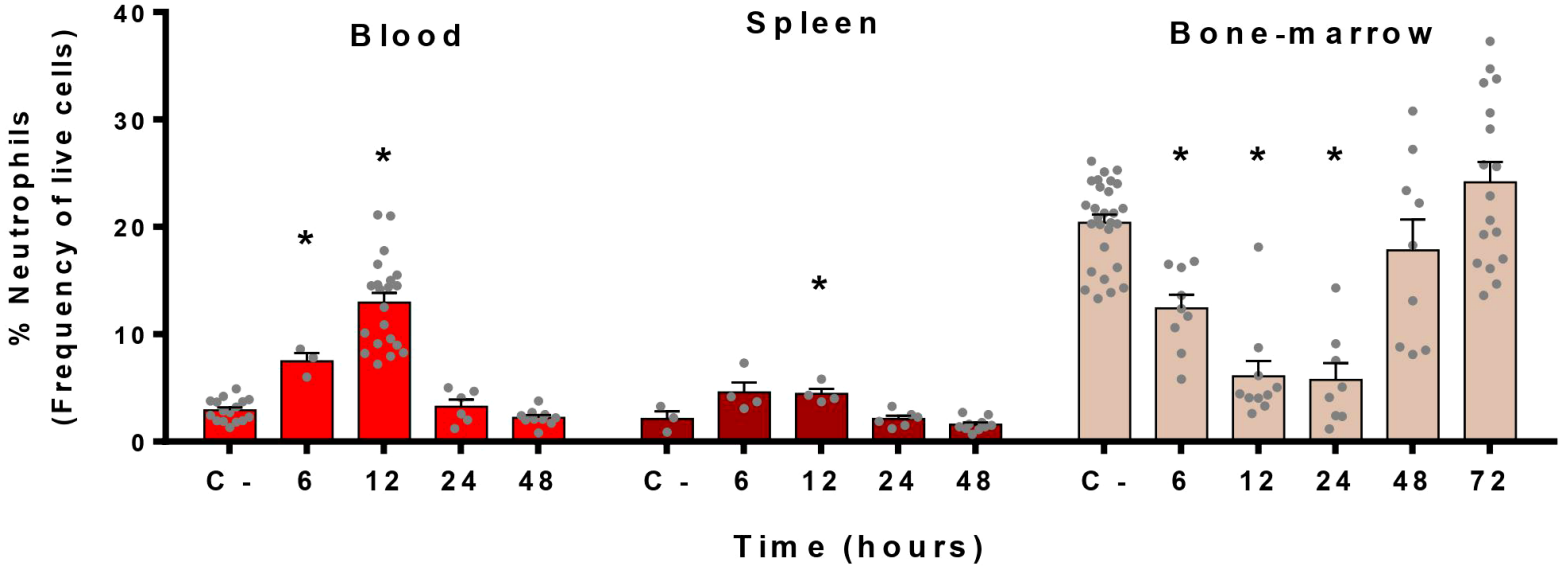
Neutrophils migrate from the bone marrow to the blood during *S. suis* infection. Mice were intraperitoneally infected with *S. suis* serotype 2 (1 x 10^7^ CFU/ml) and the blood, spleen and bone marrow were collected at different time points to analyze cells by flow cytometry. C- represents mock-infected mice (all time points pooled). Data shows the percentage of neutrophils among live cells in the indicated organs expressed as mean +/- SEM (*n =* 3 to 28). *P < 0.05, indicates a significant difference between C- and *S. suis-*infected mice.

Since neutrophils are recruited in the blood and in the spleen during the infection, we characterized the kinetics of neutrophil mobilization in these organs as well as in the bone marrow, the headquarter of neutrophil production (38). Flow cytometry analyses revealed that neutrophil rates in the blood and spleen of mice increased as soon as 6 h post-infection (p.i.) and peaked at 12 h p.i. (**Figure 2**). A return to basal level was observed after 24 h of infection. In parallel, the bone marrow, in which neutrophils represent 20% of all leukocytes during homoeostasis, showed an important drop in percentages of neutrophils from 6 h to 24 h p.i. (**Figure 2**). From 48 h onwards, bone-marrow replenished its stock in neutrophils, returning to basal levels.

### 3.3 *S. suis* infection causes mobilized neutrophils to change their phenotype

**Figure 3:**
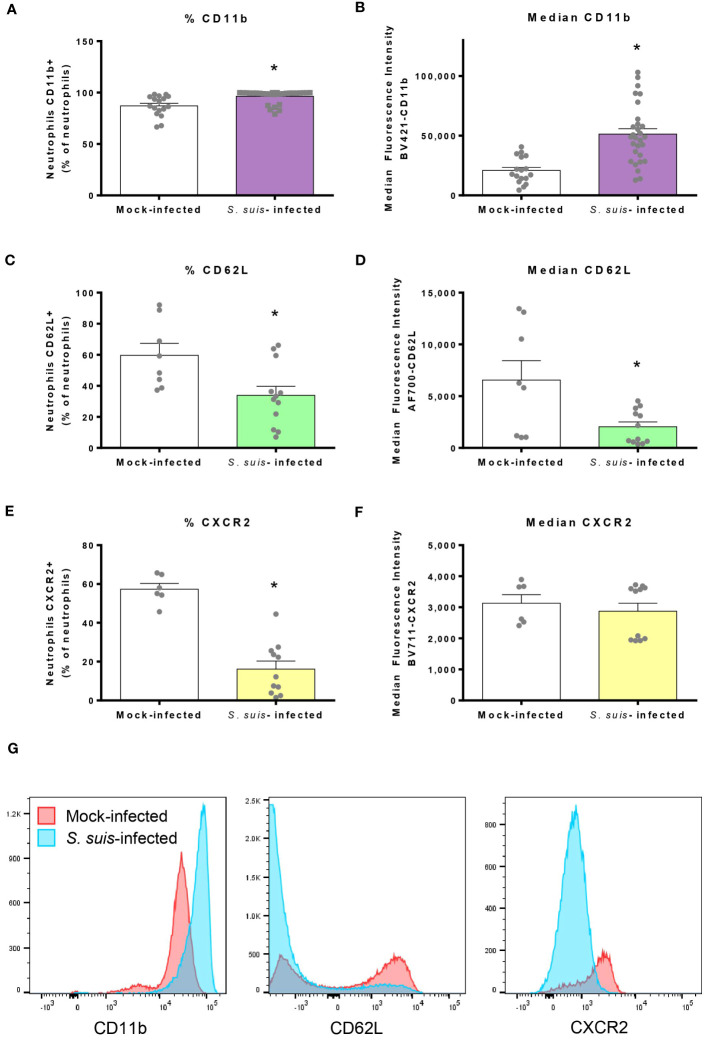
Neutrophils mobilized in the blood during *S. suis* infection change the expression of surface markers. Mice were infected intraperitoneally with 1 x 10^7^ CFU/ml of *S. suis* serotype 2. At 16 h post-infection, blood was collected to analyze markers (CD11b, CD62L and CXCR2) at the surface of neutrophils by flow cytometry. Blood neutrophils were analyzed for the percentage of cells [mean +/- SEM (*n =* 6 to 30)] expressing the indicated marker **(A, C, E)** and the median fluorescence intensity (+/- SEM) for the indicated marker **(B, D, F)**. *P < 0.05, indicates a significant difference between mock-infected and *S. suis-*infected mice. **(G)** Representative histograms of the surface markers expressed by blood neutrophils in mock-infected or *S. suis-*infected animals.

During infection, neutrophils undergo phenotypical changes to better respond to pathogen invasion (39). Most of the blood neutrophils expressed CD11b in steady state, but *S. suis* infection significantly increased this percentage from 85% to 96% (**Figures 3A, G**). In addition, neutrophils from infected mice presented higher levels of CD11b expression at their surface, as determined by the median fluorescence intensity (**Figure 3B**). On the contrary, infection reduced the percentage of neutrophils expressing CD62L and CXCR2 (**Figures 3C, E, G**). For CD62L, positive cells exhibited lower levels of molecule surface expression during the infection (**Figure 3D**), while expression levels remained unchanged for CXCR2^+^ cells (**Figure 3F**). An increased expression of CD11b was also observed in spleen neutrophils from infected mice. However, no statistical difference was observed in the percentage or expression levels of the other markers in spleen neutrophils during *S. suis* infection compared to controls. Nevertheless, the overall pattern of marker expression was similar between blood and spleen (**Supplementary Figure S2**). To compare the effect of a mild infection and a severe infection on neutrophil phenotype, we infected mice with a low dose (1 x 10^6^ CFU) or a high dose (1 x 10^7^ CFU, the dose used throughout this study) of *S. suis*. However, the phenotype of neutrophils was overall similar between the two doses (**Supplementary Figure S3**).

### 3.4 *S. suis* infection results in a massive production of G-CSF in mice

**Figure 4:**
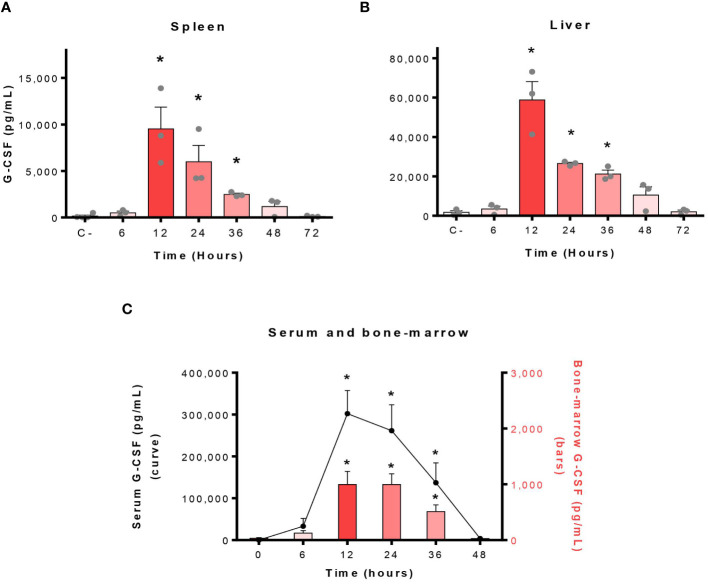
Infection by *S. suis* results in massive production of granulocyte colony-stimulating factor (G-CSF) in mice. The blood and organs of *S. suis-*infected mice (1 x 10^7^ CFU/ml; intraperitoneally) were collected at different times and G-CSF analyzed in the spleen **(A)** and liver **(B)** by Luminex, or in the serum and bone marrow **(C)** by ELISA. C- refers to mock-infected mice (all time points pooled). The data represent the mean +/- SEM (*n* = 3 to 12). *P < 0.05, indicates a significant difference between C- and *S. suis-*infected mice.

Among the factors produced during infections, the G-CSF represents the key cytokine involved in the control of neutrophils (21). Thus, we investigated if the G-CSF was produced in the serum and organs of *S. suis*-infected mice at different times post-infection. Important levels of G-CSF were produced in the spleen, the liver, the serum and the bone-marrow of infected mice with a peak of production at 12 h p.i. (**Figure 4**). Specifically, serum concentrations of G-CSF represented the highest of all evaluated cytokines at this time point (**Supplementary Figure S4**).

### 3.5 Blood leukocytes participate in G-CSF production during *S. suis* infection

**Figure 5:**
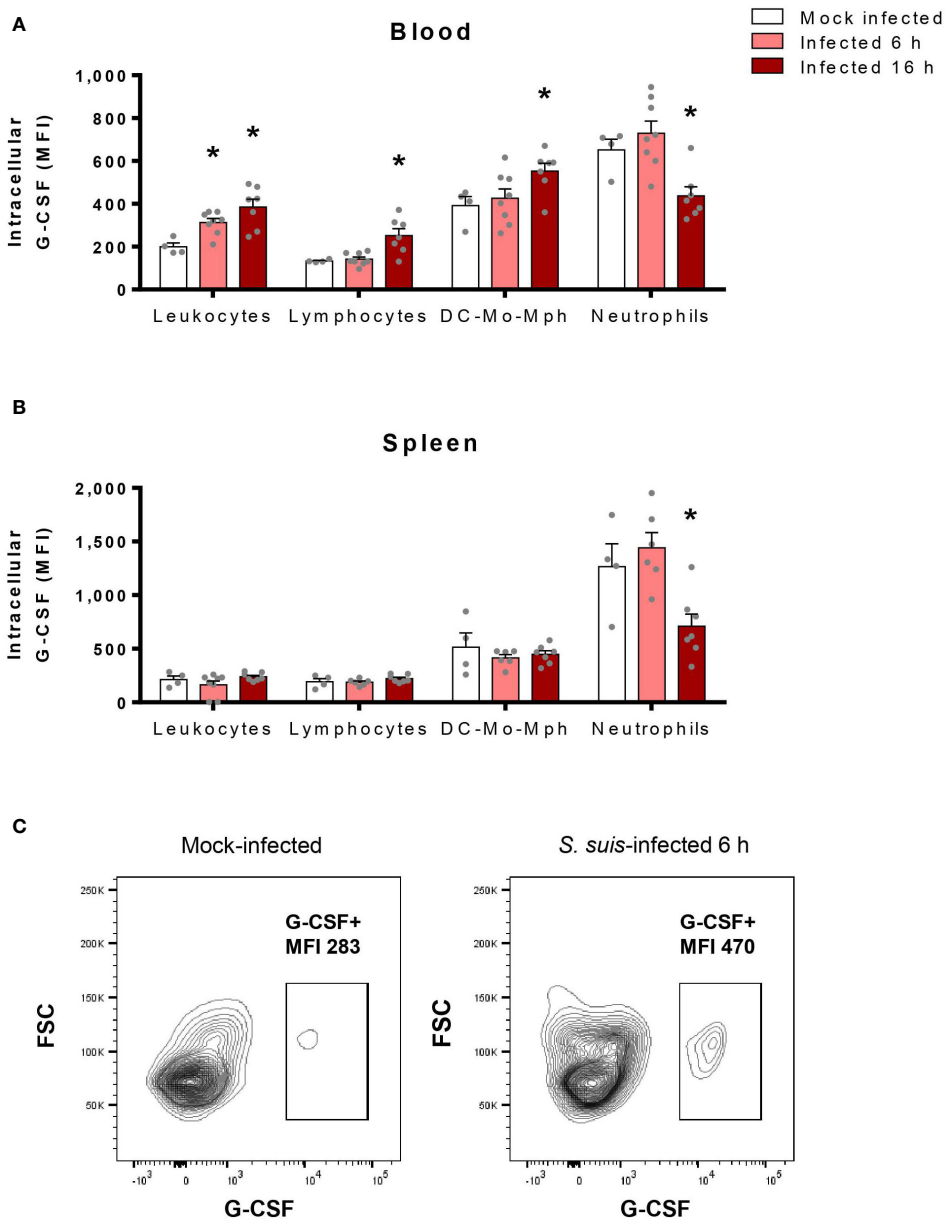
Blood hematopoietic cells produce G-CSF in response to *S. suis* infection, while neutrophils probably release their intracellular stock of G-CSF. Blood **(A)** and spleen **(B)** were collected from *S. suis*-infected (1 x 10^7^ CFU/ml; intraperitoneal) or mock-infected mice at 6 h or 16 h post-infection. The cells were stained with surface antibodies to identify populations, permeabilized for intracellular staining of G-CSF and analyzed by flow cytometry. Graphs show the median fluorescence intensity (MFI) for G-CSF staining among each population. DC-Mo-Mph stands for the pool of dendritic cells, monocytes, and macrophages. Data represent the mean +/- SEM (*n* = 4 to 8). *P < 0.05, indicates a significant difference between mock-infected and *S. suis-*infected mice. **(C)** Representative plots of the G-CSF+ population among total leukocytes in the blood of mock-infected (left) or *S. suis*-infected (right) mice after 6 h.

The massive production of G-CSF during *S. suis* infection indicates that some cells, somehow, produce this factor. Since dendritic cells (DCs) and macrophages produce G-CSF *in vitro* when stimulated with *S. suis* (30), we hypothesized that immune cells could take part in G-CSF production *in vivo*. By quantifying intracellular G-CSF in immune cells, we found that G-CSF increases in blood leukocytes in response to *S. suis* infection (**Figures 5A, C**). Particularly, this rise occurs among both lymphocytes and the pool of DCs, monocytes, and macrophages. However, neutrophils, which express the highest levels of G-CSF at the basal state, decreased their intracellular levels during infection (**Figure 5A**). In the spleen, none of the hematopoietic cells showed an increase of intracellular G-CSF, but intracellular G-CSF decreased among the neutrophil population at 16 h p.i. (**Figure 5B**).

### 3.6 G-CSF controls the release of neutrophils from the bone marrow to the blood during *S. suis* infection

**Figure 6:**
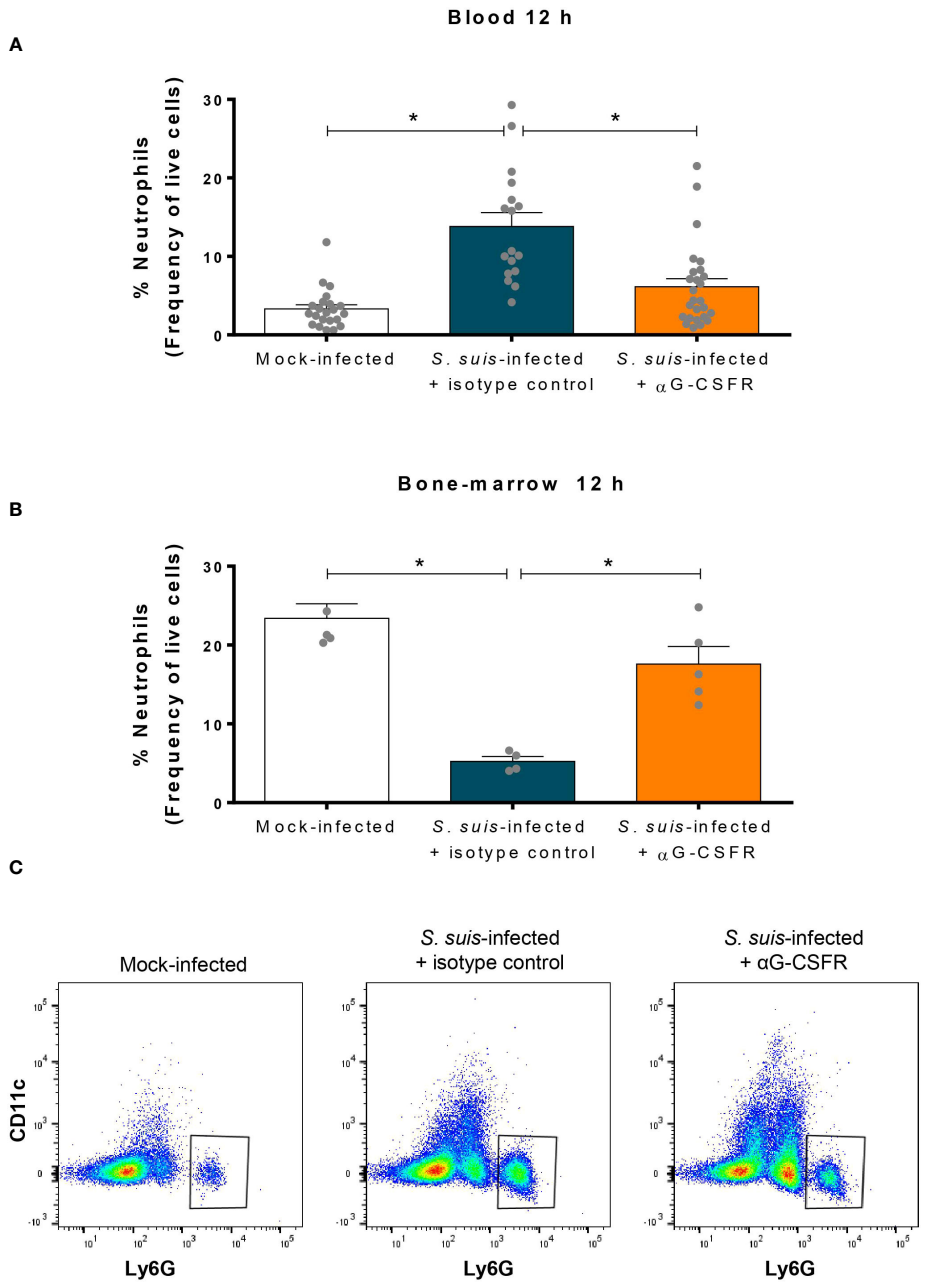
Granulocyte colony-stimulating factor (G-CSF) controls release of the neutrophils from the bone marrow to the blood during *S. suis* infection. To inhibit the effect of G-CSF *in vivo*, mice were treated with 100 µg of an antibody directed against the G-CSF receptor (αG-CSFR) or an isotype control. Mice received two doses of antibodies at day -1 and day 0 prior to intraperitoneal infection with *S. suis* serotype 2 (1 x 10^7^ CFU/ml). Blood **(A)** and bone marrow **(B)** were collected at 12 h post-infection to quantify neutrophils by flow cytometry. Data represent the mean +/- SEM (*n* = 4 to 27). *P < 0.01, indicates a significant difference between the groups connected by a line. **(C)** Representative dot plots of the blood neutrophil population in mock-infected (left) or *S. suis*-infected for 12 h treated with isotype control (middle) or αG-CSFR (right).

Since G-CSF controls neutrophil recruitment (20), we neutralized its effect in a mouse model using an anti-G-CSFR antibody (αG-CSFR) prior to *S. suis* infection (**Figure 6**). At 12 h p.i., treatment with αG-CSFR prevented neutrophil recruitment in the blood, exhibiting similar levels to those of control mice (mock-infected) (**Figures 6A, C**). Simultaneously, the drop of the neutrophil percentage in the bone marrow caused by the infection did not occur during the G-CSF receptor blockade (**Figure 6B**). The stock of neutrophils was therefore maintained in the bone-marrow under this condition. The analysis of the different leukocyte populations revealed that mainly neutrophil population was affected by G-CSFR blockade (**Supplementary Figure S5**).

### 3.7 G-CSF does not improve bacterial clearance during *S. suis* infection

**Figure 7:**
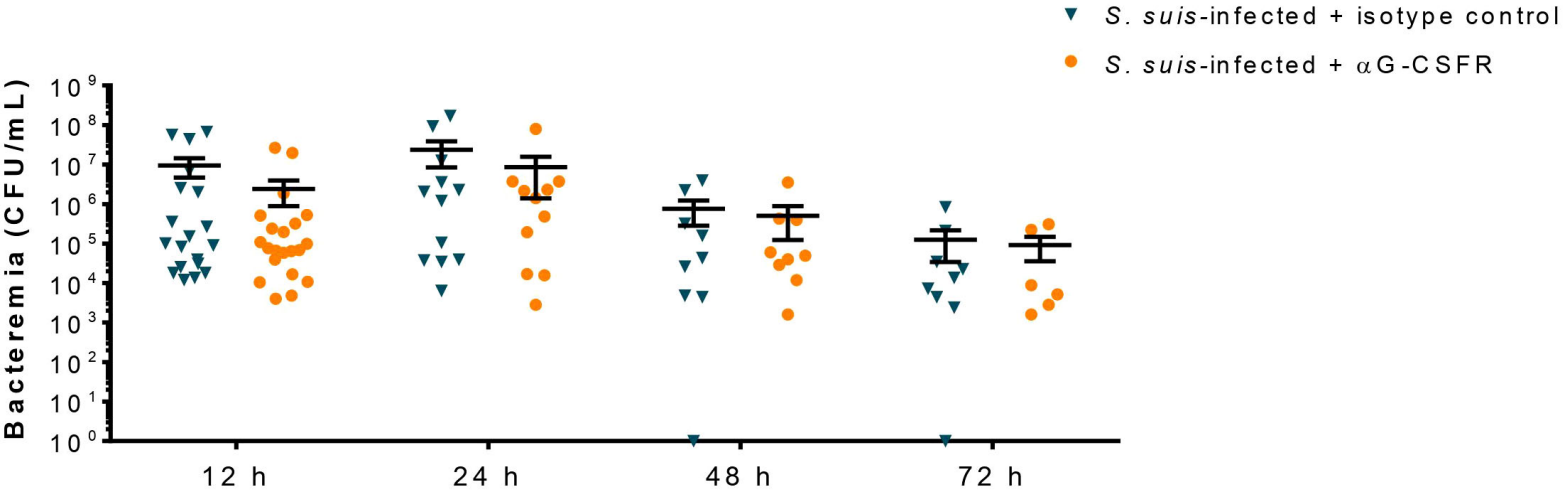
The granulocyte colony-stimulating factor (G-CSF) does not participate in *S. suis* clearance during the infection. Mice were treated with 100 µg of the antibody anti-G-CSFR (αG-CSFR) or the isotype control as described in Materials and Methods. They were then infected with *S. suis* serotype 2 (1 x 10^7^ CFU/ml; intraperitoneally). At different time points, blood was collected and the bacteremia evaluated. Each point corresponds to the colony forming unit (CFU) of bacteria per ml recovered from an individual mouse, with the black bars representing the mean +/- SEM (*n* = 6 to 21).

Since G-CSF controls neutrophil egress from the bone marrow to the blood (**Figure 6**) and neutrophils promote host survival by controlling *S. suis* bacterial burden (10), we evaluated the role of the G-CSF in *S. suis* clearance from the blood. Results showed that the treatment with αG-CSFR did not affect the concentration of bacteria in the blood of *S. suis*-infected mice at any evaluated time (**Figure 7**).

### 3.8 Role of G-CSF during the cytokine storm caused by *S. suis*

**Figure 8:**
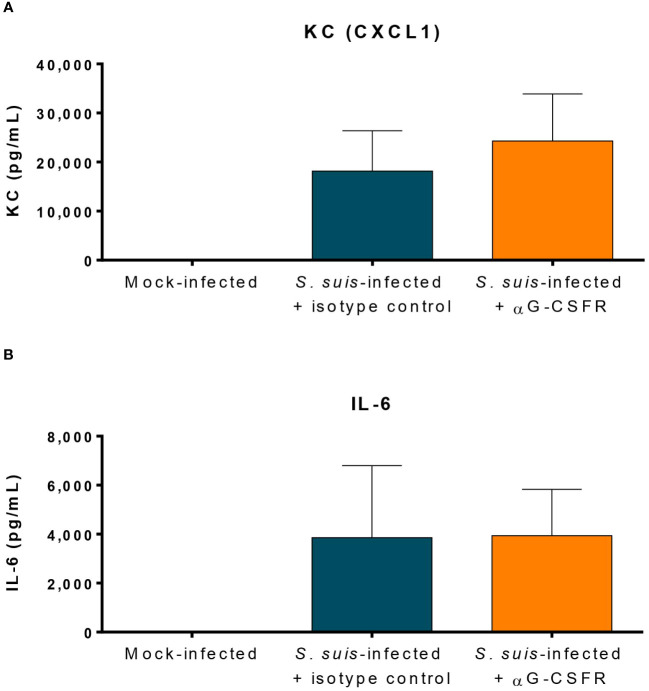
Granulocyte colony-stimulating factor (G-CSF) does not modulate the production of pro-inflammatory cytokines during *S. suis* infection. To inhibit the effect of G-CSF *in vivo*, mice were treated with 100 µg of an antibody directed against the G-CSF receptor (αG-CSFR) or the isotype control. Mice received two doses of antibodies at day -1 and day 0 prior to intraperitoneal infection with *S. suis* serotype 2 (1 x 10^7^ CFU/ml). At 12 h post-infection, blood was collected from the submandibular vein and the plasma analyzed by ELISA to quantify the keratinocyte chemoattractant/chemokine (C-X-C motif) ligand 1 (KC/CXCL1) **(A)** and the interleukin 6 (IL-6) **(B)**. The data represent the mean +/- SEM (*n* = 12 to 36).

Neutrophils participate in the production of inflammatory mediators in systemic infection (10). Since G-CSFR blockade induced less neutrophil release from the bone marrow, there might also be an impact on the levels of pro-inflammatory mediators produced during inflammation. We quantified CXCL1 (KC) and IL-6 in the plasma of *S. suis*-infected mice at 12 h p.i., time at which most cytokine levels peak (40, 41). However, G-CSFR blockade did not significantly change levels induced by *S. suis* infection (**Figure 8**). We performed additional cytokine analyses by Luminex and showed that αG-CSFR treatment did not significantly modulate the plasmatic levels of most of the cytokines (despite an upward trend), but increased IFN-γ and MIP-1α production (**Supplementary Figure S6**).

## 4 Discussion

The bacterial pathogen *S. suis* causes major economic losses to the swine industry. Despite years of research in the field, uncertainties persist regarding how the immune system handles the bacterium. Herein, we proposed to study the recruitment of neutrophils during *S. suis* infection – from its establishment to its consequences, since these cells first respond to microbial invasions. We hypothesized that *S. suis* infection causes a rapid mobilization of neutrophils under the influence of G-CSF, thereby participating in inflammation and bacterial clearance.

Our results showed that neutrophil rates rise in both the blood and the spleen during the first hours of *S. suis* infection, in a time frame similar to those reported in other infection models (7, 27, 42, 43). Following a strict contrasting pattern, the percentage of neutrophils in the bone marrow decreases during the course of *S. suis* infection. Although other studies already described neutrophil increase in the blood in response to *S. suis* (16, 17), this work is the first to characterize neutrophil changes in the spleen and bone marrow. Results suggest that the neutrophils recruited in the blood and spleen come from the bone marrow, as was reported in the context of other infections, including *Pseudomonas aeruginosa, Listeria monocytogenes* and cecal ligation and puncture-induced sepsis (7, 27, 42). Thus, we showed that a strong immune response takes place in the first hours of *S. suis* infection. In parallel to this neutrophilia, *S. suis* causes a lymphopenia and an increase in the pool of DCs, monocytes and macrophages. The lymphopenia, frequently observed during infections, may be explained by the redistribution of the lymphocytes to the lymphoid tissue to engage the adaptative immune response (44–46), their distribution to other tissues or apoptosis (47). The increased proportion of DCs, monocytes and macrophages might be due to monocyte recruitment in the blood, which participate in bacterial clearance, as reported (48, 49). However, theses hypotheses remain to be confirmed by further investigations.

Since the inflammation initiated by an infection might activate neutrophils, we evaluated surface molecule changes on the cells, as a reflection of their activation (12, 50). In *S. suis*-infected mice, blood neutrophils show an increased expression of CD11b, the shedding of the L-selectin CD62L and less cells were positive for CXCR2 expression, suggesting an activated phenotype for the neutrophils during the infection (13, 51). This first description of neutrophil phenotype during *S. suis* infection corroborates the observations made in other infection models, including in a mouse model of local *Staphylococcus aureus* infection and a primate model of simian immunodeficiency virus infection (52, 53). Similar results were observed *in vitro* with neutrophils stimulated with purified lipoproteins from *Mycobacterium tuberculosis* (51). Overall, our results indicate that *S. suis* infection do not alter the activated phenotype of blood neutrophils. Given the activated phenotype of neutrophils in the blood of *S. suis*-infected mice, exploring their *in vivo* functionality becomes imperative to gauge their effector potential in this model.

Neutrophil mobilization and activation depend on the inflammatory environment, including the effect of some pro-inflammatory factors. Among them, G-CSF seems to be critical for neutrophil control (21). Limited information exists on G-CSF production in the context of *S. suis* infection; however, we recently detected high levels of G-CSF in the plasma of *S. suis*-infected mice (30). We demonstrated that G-CSF is also detectable in the spleen and the liver of infected mice, the major filtration organs, as well as in the bone marrow, the source of neutrophil production. Moreover, we showed that the amounts of G-CSF produced in the plasma during *S. suis* infection exceed by far the levels of other cytokines, suggesting that G-CSF production constitutes an important feature of the immune response against *S. suis*. A previous work studied G-CSF production *in vitro* and showed that dendritic cells and macrophages produce G-CSF in response to *S. suis* stimulation (30). Our *in vivo* experiments showed that, in the blood, lymphocytes and the pool of DCs, monocytes and macrophages produce G-CSF throughout the infection, suggesting that they contribute to the high plasmatic levels observed in *S. suis*-infected mice. However, none of the spleen hematopoietic cells produced G-CSF in response to *S. suis* infection, foreshadowing the implication of other cell types in local production. Although neutrophils express the most G-CSF at a basal state, infection reduced their intracellular expression of G-CSF in both the blood and the spleen. We thus hypothesize that neutrophils stock G-CSF in their granules and release it during the infection, but this remains to be demonstrated. Non-hematopoietic cells can also produce G-CSF as demonstrated by Boettcher et al. (54), who identified *in vivo* that endothelial cells mainly produce G-CSF during a LPS challenge in mice. Thus, evaluating G-CSF production by endothelial cells or other non-hematopoietic cells could be a next step to study G-CSF production in the context of *S. suis* infection.

As previously mentioned, the impressive levels of G-CSF produced during *S. suis* infection might reflect its preponderant role during the infection. Therefore, we neutralized the G-CSFR with an antibody to investigate the role of G-CSF in neutrophil recruitment, bacterial clearance, and production of pro-inflammatory mediators – which characterize *S. suis* infection. Our results demonstrated for the first time that G-CSF causes the egress of neutrophils from the bone marrow to the blood during *S. suis* infection, supporting the observations made in other infection models (26–28, 55). Of note, most studies used knock-out mice models for G-CSF or its receptor, whose phenotypes present an altered basal production of neutrophils. Our model, using a neutralizing antibody, prevented this bias, with no alteration of neutrophil production in steady state.

Since neutrophils were recently shown to reduce bacteremia and produce cytokines during *S. suis* infection (10), we expected G-CSF-recruited neutrophils to exhibit the same characteristics. Surprisingly, G-CSF production does not impact bacteremia in our model, conflicting with other infection models in which G-CSF improved pathogen clearance (26–28, 56). However, our results corroborate those obtained in an infection model of *Streptococcus pneumoniae*, in which G-CSF does not affect bacterial load in the lungs at 48 h p.i (55, 57). Since bacteria colonize various organs of the infected host, it could be interesting to study if G-CSF-recruited neutrophils infiltered in the tissue better control bacterial load in the organs. Our data also showed that G-CSF does not significantly affect the production of pro-inflammatory mediators induced by *S. suis*, except for IFN-γ and MIP-1α, which were enhanced by G-CSF neutralization (**Supplementary Figure S6**). These two cytokines activate and recruit immune cells, particularly monocytes and macrophages. Reports in the literature suggest that G-CSF defects enhance cytokine production in response to various pathogens, with immune cells overproducing other cytokines to compensate for G-CSF neutralization, or that G-CSF could regulate cytokine production (26, 27, 56). Both the method to neutralize the G-CSF (antibody vs. knock-out) and the features of the studied pathogen might explain the differences observed. As such, more studies are required to untangle the mechanisms behind the pathogen-dependent effect of G-CSF on clearance and inflammatory mediator production. In our model, antibody-mediated neutralization of G-CSF did not significantly influence bacterial clearance and cytokine production during *S. suis* infection. A possible hypothesis would be that the “steady-state” neutrophils (the ones already in circulation before the G-CSF-driven recruitment) and the other immune cells can compensate for the altered G-CSF-driven recruitment of neutrophils.

## 5 Conclusion

**Figure 9:**
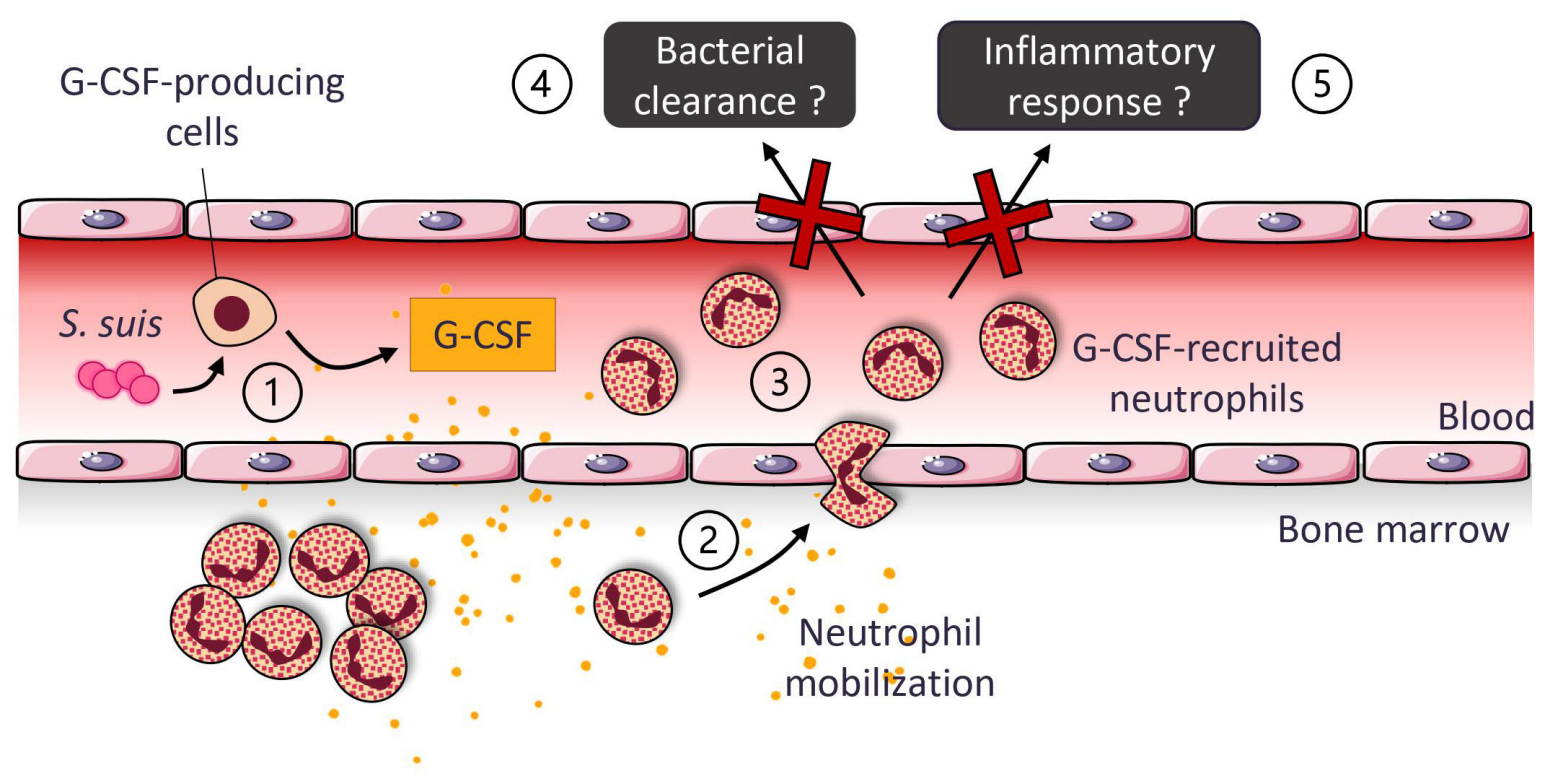
Suggested model for the production and role of the granulocyte colony-stimulating factor (G-CSF) during *S. suis* infection. *S. suis* infection induces a systemic production of G-CSF by blood leukocytes and possibly other cell types ①. G-CSF causes the release of bone marrow neutrophils into the blood, increasing the percentage of those cells in circulation ②. Blood neutrophils express activation markers ③. However, G-CSF-recruited neutrophils do not significantly participate in bacterial clearance ④ or inflammatory response ⑤.

We found that *S. suis* infection induces neutrophil recruitment and activation in the blood. Neutrophils egress from the bone marrow to the blood under the influence of G-CSF, a cytokine highly produced in response to the bacteria (**Figure 9**). This research provides insight into neutrophil recruitment and role during *S. suis* infection. Although G-CSF controls neutrophil recruitment, it is not crucial for bacterial clearance and cytokine production, suggesting that compensatory mechanisms exist within the immune system when G-CSF is neutralized. The progresses in fundamental knowledge constitute the groundwork for therapeutical avenues, with this study supporting the idea that the prophylactic administration of G-CSF shows limited benefits, as already evocated by Brockmeier et al. (29).

## Data availability statement

The original contributions presented in the study are included in the article/**Supplementary Material**. Further inquiries can be directed to the corresponding author.

## Ethics statement

The animal study was approved by Animal Welfare Committee of the University of Montreal. The study was conducted in accordance with the local legislation and institutional requirements.

## Author contributions

MB: Writing – review & editing, Writing – original draft, Visualization, Validation, Project administration, Investigation, Formal Analysis, Conceptualization. ML: Methodology, Writing – review & editing, Investigation, Formal Analysis. J-PA: Writing – review & editing, Investigation, Formal Analysis, Conceptualization. MG: Writing – review & editing, Supervision, Resources, Funding acquisition, Formal Analysis, Conceptualization. MS: Writing – review & editing, Visualization, Supervision, Resources, Project administration, Investigation, Funding acquisition, Formal Analysis, Conceptualization.
