# Supplementary material for: The granulocyte colony-stimulating factor produced during Streptococcus suis infection controls neutrophil recruitment in the blood without affecting bacterial clearance
Source: Front Immunol. 2024 Aug 2;15:1403789. doi: 10.3389/fimmu.2024.1403789 (PMC11327821; doi:10.3389/fimmu.2024.1403789)
Supplement: Supplementary file 1 [file DataSheet_1.docx]

Supplementary Material

##
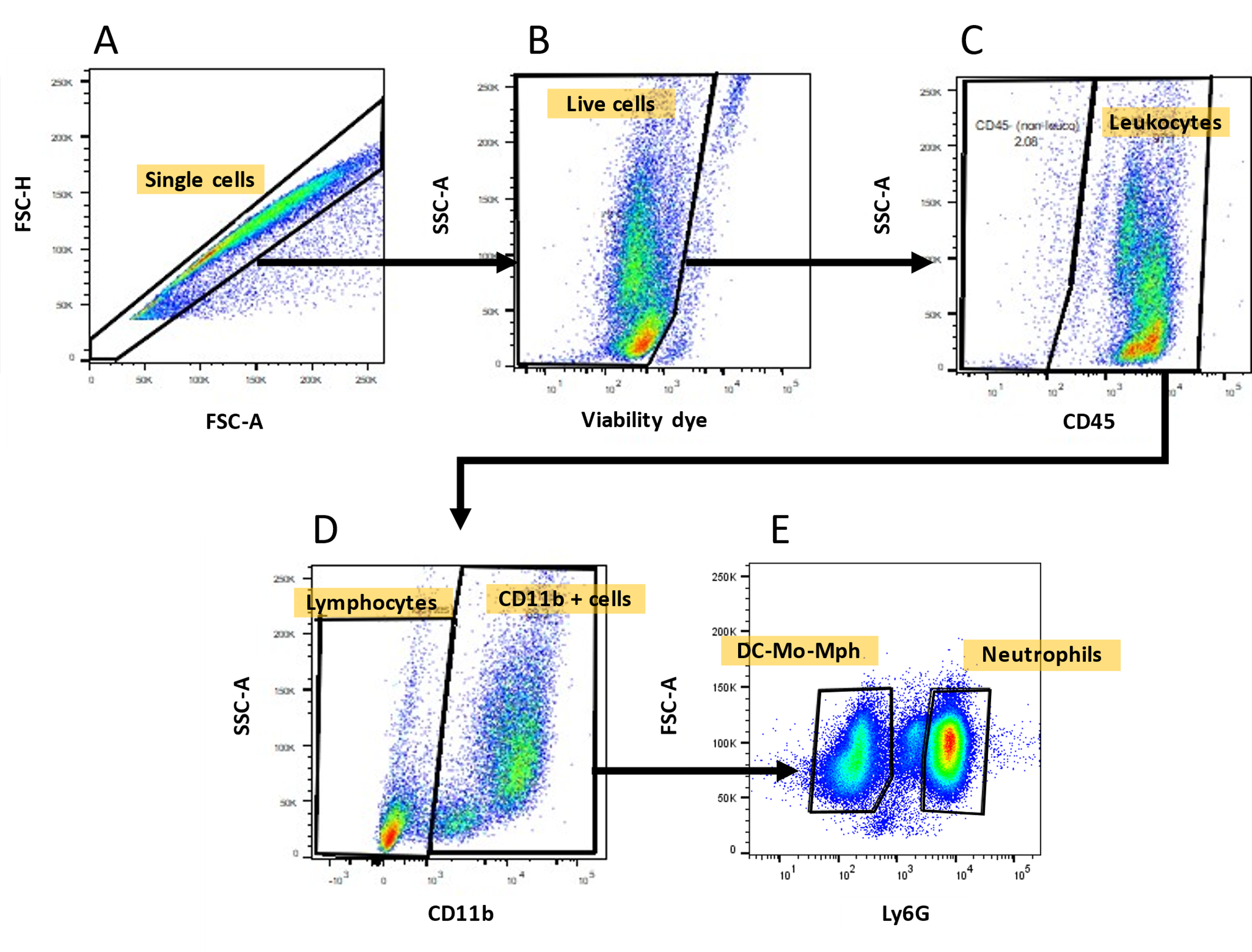
Supplementary Figures

**Supplementary Figure 1. Flow cytometry gating strategy used to define immune cell populations in the blood, spleen and bone marrow**. Representative analysis of the blood of a mouse infected with *S. suis*. Single cells were gated to exclude doublets from the analysis (**A**). Dead cells were excluded using a viability dye (**B**). Total leukocyte population was gated as CD45+ cells (**C**). Lymphocytes were gated as CD11b- cells, while CD11b+ cells were further analyzed with Ly6G marker (**D**). CD11b+ Ly6G- cells were identified as the pool of dendritic cells (DC), monocytes (Mo) and macrophages (Mph) while CD11b+ Ly6G+ cells were identified as neutrophils (**E**).

**Supplementary Figure 2.** **The neutrophils mobilized in the spleen during *S. suis* infection change the expression of surface markers**. Mice were infected intraperitoneally with 1 x 10^7^ CFU/ml of *S. suis* serotype 2 strain P1/7. At 16 h post-infection, spleens were collected to analyze the markers (CD11b, CD62L and CXCR2) at the surface of the neutrophils by flow cytometry. Spleen neutrophils were analyzed for the percentage of cells expressing the indicated marker (**A, C, E**) and the median fluorescence intensity for the indicated marker (**B, D, F**). * *P* < 0.05, indicates a significant difference between mock-infected and *S. suis-*infected mice (*n* = 6 to 19).

**Supplementary Figure 3. The infectious dose does not impact the phenotype of neutrophils mobilized during *S. suis* infection**. Mice were infected intraperitoneally with 1 x 10^6^ (low-dose) or 1 x 10^7^ (high dose) CFU/ml of *S. suis* serotype 2 strain P1/7. At 16 h post-infection, blood and spleens were collected to analyze the markers (CD11b, CD62L and CXCR2) at the surface of the neutrophils by flow cytometry. Neutrophils were analyzed for the percentage of cells expressing the indicated marker (**A, C, E**) and the median fluorescence intensity for the indicated marker (**B, D, F**) (*n* = 6 to 19). * *P* < 0.05, indicates a significant difference between mock-infected and *S. suis-*infected mice or between doses (when indicated with a line).

**Supplementary Figure 4.** **G-CSF is present in higher amounts than other cytokines in the plasma of *S. suis*-infected mice.** Blood of *S. suis-*infected mice (1 x 10^7^ CFU/ml; intraperitoneal) was collected at 12 h and the cytokines analyzed in the serum by Luminex. The data represent the mean +/- SEM (*n* = 4).

**Supplementary Figure 5.** **G-CSFR inhibition mainly affects neutrophil population.** To inhibit the effect of G-CSF *in vivo*, mice were treated with 100 µg of an antibody directed against the G-CSF receptor (αG-CSFR) or an isotype control. Mice received two doses of antibodies at day -1 and day 0 prior to intraperitoneal infection with *S. suis* serotype 2 (1 x 10^7^ CFU/ml). Blood (**A**) and bone marrow (**B**) were collected 12 h post-infection to count immune cells by flow cytometry. DC-Mo-Mph stands for the pool of dendritic cells, monocytes, and macrophages. Data represent the mean +/- SEM (*n* = 6 to 11). * *P* < 0.05, indicates a significant difference between the groups connected by a line.

**Supplementary Figure 6.** **G-CSF significantly modulates the production of few pro-inflammatory cytokines and chemokines during *S. suis* infection*.*** To inhibit the effect of G-CSF *in vivo*, mice were treated with 100 µg of an antibody directed against the G-CSF receptor (αG-CSFR) or an isotype control. Mice received two doses of antibodies at day -1 and day 0 prior to intraperitoneal infection with *S. suis* serotype 2 (1 x 10^7^ CFU/ml). At 12 h post-infection, blood was collected from the submandibular vein and the plasma analyzed by Luminex for pro-inflammatory mediator quantification. The data represent the mean +/- SEM (*n* = 3 to 20). * *P* < 0.05, indicates a significant difference between isotype control and αG-CSFR treated mice.
